# T cell knockout attenuates HFD-induced increases in blood pressure in female and male Dahl rats

**DOI:** 10.1042/CS20257273

**Published:** 2025-09-09

**Authors:** Lindsey A. Ramirez, Elizabeth Snyder, Riyaz Mohamed, Justine M. Abais-Battad, Hannah R. Godley-Boswell, John Henry Dasinger, David L. Mattson, Mike W. Brands, Babak Baban, Ahmed Elmarakby, Michael J. Ryan, Jennifer C. Sullivan

**Affiliations:** 1 Physiology, Medical College of Georgia at Augusta University, Augusta, GA, U.S.A.; 2 Oral Biology, Medical College of Georgia at Augusta University, Augusta, GA, U.S.A.; 3 Columbia VA Health Care System, Columbia, SC, U.S.A.; 4 University of South Carolina School of Medicine, Columbia, SC, U.S.A.

**Keywords:** inflammation, mean arterial pressure, sex, telemetry

## Abstract

Increased body weight is associated with a higher incidence of hypertension in humans. Pre-clinical evidence shows that a high-fat diet (HFD) promotes hypertension. While T cells have been implicated in hypertension, the contribution of T cells to HFD-induced increases in blood pressure (BP) remains unknown. We tested the hypothesis that T cells mediate HFD-induced increases in BP in female and male Dahl rats. Female and male wildtype (WT) Dahl rats were randomized to a normal-fat diet (0.16% kcal from fat) or HFD (59% kcal from fat) from 5 to 15 weeks of age. Aortic and renal T cells were measured by flow cytometry. Additional female and male WT and CD247 (CD3ζ-chain) knockout (KO) Dahl rats were placed on a HFD from 5 to 15 weeks of age. Body weight and fat and lean mass were measured throughout, and BP was measured by telemetry throughout the last five weeks of treatment. At 15 weeks of age, rats were euthanized, and adipose tissues were weighed. HFD increased pro-inflammatory T cells and decreased anti-inflammatory T regulatory cells in the aorta and kidney in both sexes, indicating an increase in the pro-inflammatory status. HFD increased BP comparably in females and males, and the change in BP in response to HFD was blunted in CD247 KO rats of both sexes. The lower BP in CD247 KO rats was independent of changes in body weight or total fat mass. Our findings suggest that T cells contribute to HFD-induced hypertension in both sexes independent of changes in adiposity.

## Introduction

Cardiovascular disease (CVD) is the leading cause of death in women and men [1]. Hypertension is the highest-ranked risk factor in women contributing to CVD-related mortality and the second-highest in men [2]. Despite the availability of a diverse array of antihypertensive treatments, only ~21% of hypertensive patients achieve adequate blood pressure (BP) control [3]. The high proportion of patients with uncontrolled BP highlights the need to better understand mechanisms underlying the development of hypertension in both females and males. High consumption of dietary fat is linked to the development of hypertension in pre-clinical studies [4,5], and in clinical studies, decreases in weight lower the incidence of hypertension [6]. Due to the deleterious effects of a high-fat diet (HFD) on cardiovascular health, it is recommended to limit saturated fat intake to less than 10% of total calories, yet many people consume much more than this [5,7]. Thus, it is imperative to understand how a HFD affects BP.

Despite the overall lower incidence, higher awareness, and similar or better control of BP in young women vs. men, women have greater mortality due to hypertension than men (51.6% vs. 48.4%, respectively) [8]. Furthermore, controlling BP provides a greater decrease in CVD mortality in women (38%) than men (30.4%) [9]. One factor that may contribute to the increased mortality in women is adiposity, since the number of deaths attributed to high body mass index (BMI) is greater in women than in men (1.24 million vs. 1.15 million) [8,10]. Additionally, the Framingham Heart Study found that a BMI > $25\frac{kg}{m^{2}}$ was associated with 62% of hypertension cases in women, but only 34% of cases in men [11]. Thus, understanding how a HFD promotes hypertension is critical since the prevalence of severe obesity, particularly among women, continues to rise [8,12].

Inflammation is an important mediator of hypertension [13–16]. More specifically, T cells contribute to the development of hypertension in humans [17–20] and experimental animal models [13,21–26]. A genome-wide association study identified CD247 as a candidate gene for hypertension in humans [17]. CD247 encodes the CD3ζ chain of the T cell receptor [13,15]. Though studies have suggested a role of T cells in HFD-induced hypertension, and T cells are implicated in adipose inflammation associated with obesity [24,27], a direct role for T cells in HFD-induced hypertension has not been investigated in either sex.

Our laboratory and others reported that a HFD increases BP in both female and male Dahl rats [24,28–30]. While female Dahl rats fed high salt or female mice fed high fat have blunted increases in BP relative to males, female Dahl rats are not protected from HFD-induced increases in BP relative to males [24,28–30]. This makes female Dahl rats an excellent model to study enhanced BP susceptibility of females to a HFD [24,28–30]. Importantly, HFD increases BP in both female and male Dahl rats in the absence of metabolic complications such as elevated triglycerides, fasting blood glucose, cholesterol, or insulin [16,24,28]. Moreover, the HFD-induced increase in BP in Dahl rats is associated with T cell inflammation and is attenuated by immunosuppressive therapies [16,24]. The current study will directly test the hypothesis that T cells mediate HFD-induced increases in BP in Dahl rats by utilizing female and male wildtype (WT) Dahl rats and CD247 Dahl genetic knockout (KO) rats.

## Methods

### Animals

All animal experiments were approved by the Augusta University Institutional Animal Care and Use Committee and conducted in accordance with the National Institutes of Health Guide for the Care and Use of Laboratory Animals. Initial studies included female and male WT Dahl rats from a colony maintained at Augusta University originally obtained from the Medical College of Wisconsin (SS/JrHsdMcwi). Rats were maintained in temperature- and humidity-controlled rooms on a 12-hour light:dark cycle and maintained on an American Institute of Nutrition (AIN) purified diet containing 0.4% NaCl (Dyets, cat# 113755 GI). Rats were weaned at ~21 days of age and maintained on the AIN purified diet. At five weeks of age, rats were randomized to receive either a normal-fat diet (NFD) with 0.16% kcal from fat (*Bio-serv, cat# F4031, https://www.bio-serv.com/pdf/F4031.pdf*) or a HFD with 59% kcal from fat (major fat component is lard, *Bio-serv, cat # F3282, https://www.bio-serv.com/pdf/F3282_S3282.pdf*) for ten weeks. Note, both NFD and HFD contain equal amounts of NaCl, 0.4%. Food and water were available *ad libitum*.

To directly assess the contribution of T cells to HFD-induced increases in BP and adiposity, additional studies included WT female and male Dahl rats and CD247 genetic KO Dahl rats (T cell KO). Confirmation of T cell KO is shown in Supplementary Figure S1. The CD247 KO was originally obtained from the Medical College of Wisconsin. Rats were bred in the barrier facility using homozygous breeding pairs. Rats of the same strain and sex were group-housed. As above, all rats were kept on an AIN purified diet (*cat# 113755* GI) until being switched to a HFD at five weeks of age. At approximately eight weeks of age, a subset of rats was implanted with radio telemeters as previously described [13,31]. Rats were allowed one week to recover prior to collecting data. BP was measured continuously for five weeks, from week 5 to 10 of dietary treatment. N values were as follows: **WT: HFD-female** = 12, **HFD-male** = 14; **T cell KO: HFD-female** = 10, and **HFD-male** = 9.

Several litters were used in the current manuscript; not all animals were used for each measurement. N values for each experiment are included. At the end of the ten weeks of dietary treatment, all rats were euthanized between 7:00 and 9:00 am according to the recommendations of the Panel on Euthanasia of the American Veterinary Medical Association. Rats were placed in an induction chamber with a mixture of vaporized isoflurane and 95% oxygen (~1.5% isoflurane) and confirmed to be unconscious via toe pinch. A midline incision was made, animals were exsanguinated via abdominal aorta, and the diaphragm was cut before excising the heart. Tissues were collected for processing as described below.

### Quantification of T cells

**Figure 1:**
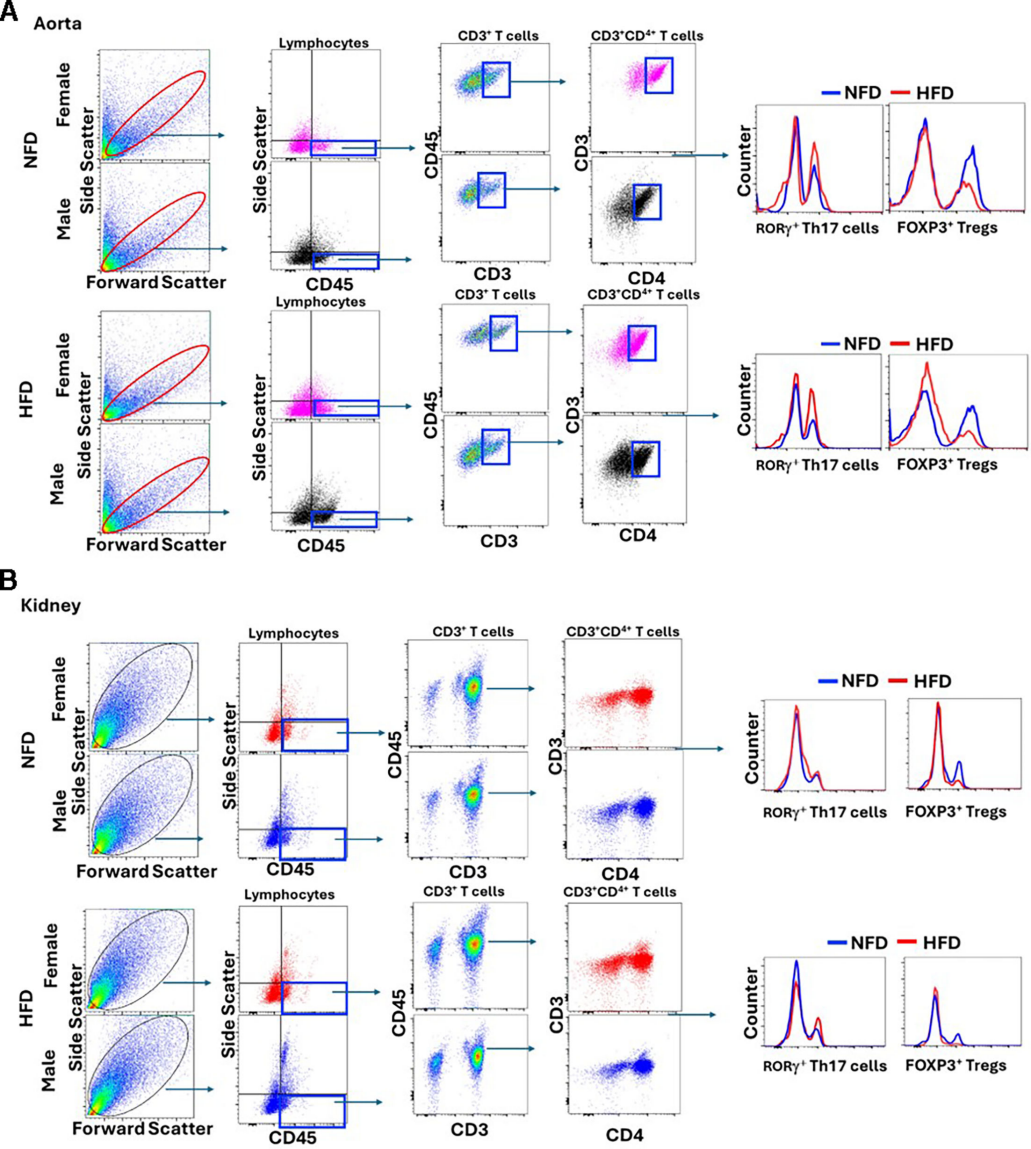
Representative flow cytometry analysis gating strategy for T cells in (**A**) aorta and (**B**) kidney. Tissues were first gated on lymphocytes, then CD3 and CD4. CD4 was further gated to Th-17 (ROR-γ) and Tregs (FoxP3). HFD, high-fat diet; NFD, normal-fat diet.

Flow cytometric analysis was performed on aorta with intact perivascular adipose tissue and kidney samples as previously described [24]. For a list of antibodies used, please refer to the *Major Resources Table in Supplemental Materials*. The same tissue was used for single-color controls. Events were collected with CytExpert Software. Compensation and gating were performed using FlowJo. Representative gating is shown in Figure 1. N values for aorta were as follows: **NFD-male** = 8, **HFD-male** = 6, **NFD-female** = 7, and **HFD-female** = 7. Note, due to an experimental error, Th17 cells were not quantifiable in three females on both NFD and HFD, resulting in *N* = 3–4. N values for kidney were as follows: **NFD-male** = 8, **HFD-male** = 8, **NFD-female** = 7, and **HFD-female** = 7.

### Measurement of body weight, body fat mass, and adipose tissue depots

Rats were weighed weekly (**WT: HFD-females** = 7, **HFD-males** = 12; **T cell KO: HFD-females** = 7, **HFD-males** = 11). At 0, 5, and 10 weeks of dietary treatment, body fat mass was measured via nuclear magnetic resonance (NMR) imaging (*Bruker minispec*). N values for the NMR data were as follows: **WT: HFD-females** = 7, **HFD-males** = 12; **T cell KO: HFD-females** = 7, **HFD-males** = 11. Note, rats with telemetry implants cannot be imaged via NMR. Rats were placed in an induction chamber and anesthetized with isoflurane. Depth of anesthesia was measured by tilting the induction chamber. Once anesthetized, the rats were gently placed in a plastic-restraint tube and placed in the NMR machine; readings lasted approximately 20 seconds. Animals were returned to home cages as they were regaining consciousness. To confirm the NMR results, following euthanasia, gonadal and peri-renal adipose tissues were isolated and weighed. N values for the adipose tissue data were as follows: **WT: HFD-female** = 7, **HFD-male** = 12; **T cell KO: HFD-female** = 4–7, **HFD-male** = 7–11. Since males are heavier than females, adipose tissue was normalized to body weight.

### Metabolic cage studies

Metabolic cage studies were conducted at baseline (week 0) and following five and ten weeks of dietary treatment in a subset of rats. Rats were acclimated to cages for ~12 hours before a 24-hour collection of urine and measurement of food consumption was performed to assess fat intake. Due to a technical error, data for kcal of fat consumed was missing for the five week time point for one HFD T cell KO female and two HFD T cell KO males. The data for baseline and ten week treatment were included for these rats. N values were as follows: **WT: HFD-female** = 4–5, **HFD-male** = 5–8; **T cell KO: HFD-female** = 5, **HFD-male** = 7–9.

The 24-hour urine samples were collected and centrifuged to separate food debris from the urine (12,000 rpm, 10 minutes, 4°C). Urinary protein was measured by incubating urine samples with Pierce BCA protein assay reagent (*ThermoFisher, cat# 23225*) at room temperature for 5 minutes. A spectrophotometer was used to measure the absorbance at 595 nm, and protein excretion (concentration × dilution × 24-hour urine volume) was calculated. N values were as follows: **WT: HFD-female** = 6, **HFD-male** = 6; **T cell KO: HFD-female** = 4, **HFD-male** = 4.

### Vascular reactivity

In a subset of rats, the thoracic aorta was harvested, cut into 2-mm rings, and mounted on pins for isometric myography (Danish Myo Technology A/S, Aarhus, Denmark) in chambers filled with aerated (95% O2 and 5% CO2) Krebs buffer (130 mM NaCl, 4.7 mM KCl, 1.17 mM MgSO_4_, 14.9 mM NaHCO_3_, 5.6 mM Dextrose, 1.56 mM CaCl_2_, and 0.03 mM EDTA) heated to 37°C. Tension was adjusted to 30 mN, and rings were allowed to equilibrate for 30 minutes with the Krebs buffer replaced every 15 minutes before the viability of each vessel was determined by a robust vasoconstrictor response to 10^-6^ M phenylephrine (PE) followed by vasorelaxation to 10^-6^ M acetylcholine (Ach). Only arteries that relaxed at least 80% of the maximal PE-induced contraction were included in the study. Cumulative endothelium-dependent relaxation to Ach (10^-9^ − 10^-5^ M) was assessed in vessel pre-constricted with PE (10^-6^). Vasorelaxation data are presented as % relaxation, as analyzed by the equation [(maximum PE response − ACh response)/(maximum PE response − baseline before PE constriction)] × 100. N values were as follows: **WT: HFD-female** = 6, **HFD-male** = 5; **T cell** **KO**: **HFD-female** = 6, **HFD-male** = 6.

### Statistical analyses

Data were analyzed with GraphPad Prism software (version 9.3.1). Flow cytometry data were compared via independent sample t-tests. Body weight, kcal of fat consumed, final mean arterial pressure (MAP), body composition, adipose tissue weight, proteinuria, and vascular reactivity were compared via two-way ANOVA followed by a Tukey’s *post-hoc* test. Data are shown as means ± SEM, and significance for all analyses was set to *P*≤0.05.

## Results

### HFD increased T cells in the aorta and kidney for both female and male Dahl rats

**Figure 2:**
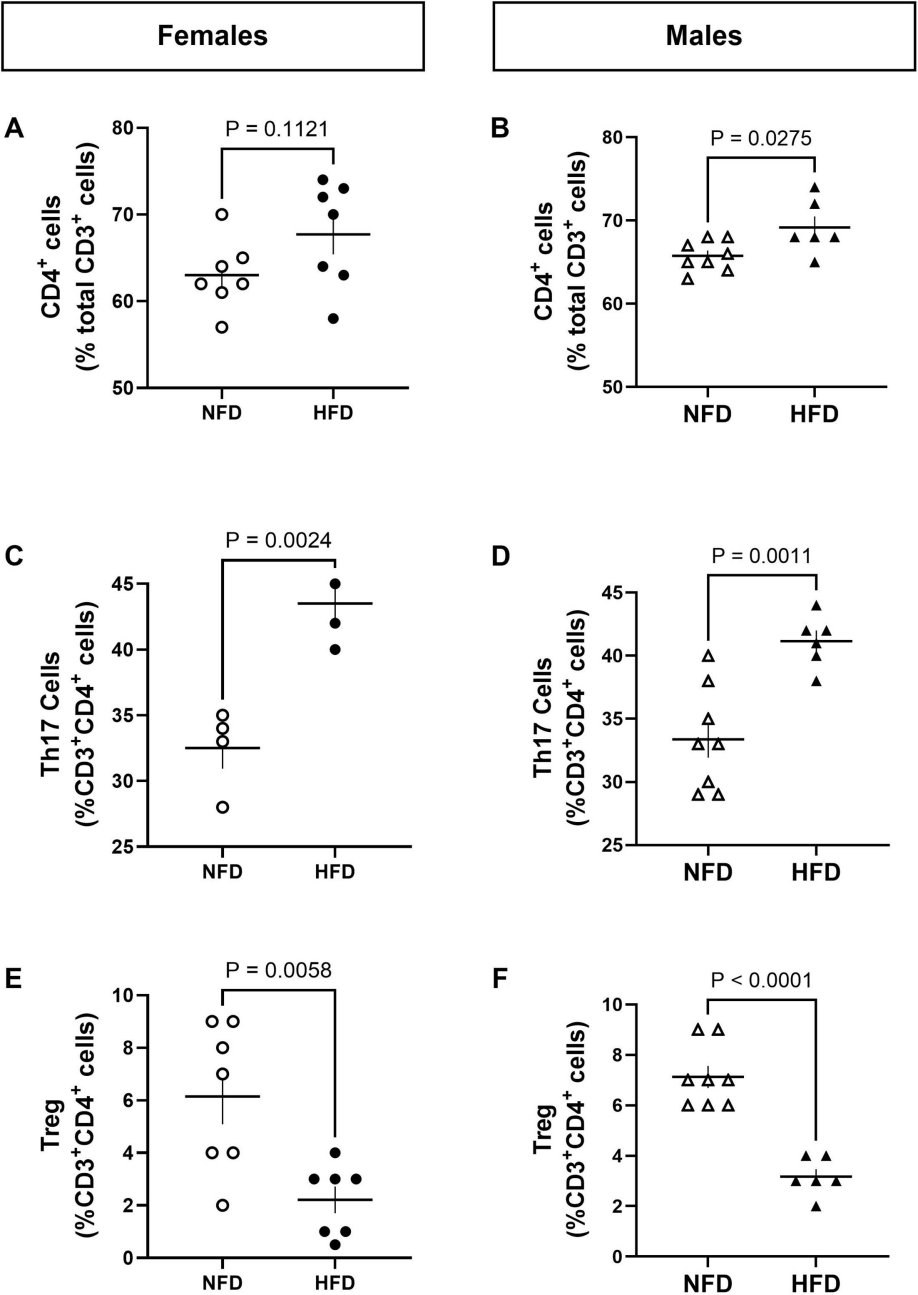
HFD promotes pro-inflammatory immune status in the aorta. Flow cytometry was used to measure the T cell profile in aorta of female (left) and male (right) Dahl WT rats following ten weeks of normal-fat diet (NFD) or high-fat diet (HFD). The percentage of (**A-B**) CD4^+^, (**C-D**) Th17^+^ cells, and (**E-F**) Foxp3^+^ Tregs were measured. N values: **NFD-female** = 7, **NFD-male** = 8, **HFD-female** = 7, **HFD-male** = 6. Note, due to an experimental error, Th17 cells were not quantifiable in three females on both NFD and HFD resulting in *N* = 3 and 4 for that T cell subtype only. Between group differences were calculated via t-tests. Values are presented as mean ± SEM. HFD, high-fat diet; NFD, normal-fat diet; Treg, T regulatory cell.

T cells were measured in the aorta by flow cytometry. The percentage of aortic CD3^+^ T cells was greater in males (**NFD-male**: 12.00 ± 0.6, **HFD-male**: 18.67 ± 0.7, P_t-test_ < 0.0001) and females (**NFD-female**: 12.00 ± 1.04, **HFD-female**: 18.86 ± 0.65, P_t-test_ <0.0001) on a HFD compared with a NFD. Total aortic T cells were gated to gain insights into subtypes. Aortic CD4^+^ T cells were significantly greater in males on a HFD vs. NFD (Figure 2B) and tended to be greater in females on a HFD (Figure 2A), but this did not reach statistical significance (*P* = 0.11). RORγ^+^ Th17 cells were greater in aorta from females (Figure 2C) and males (Figure 2D) on a HFD vs. NFD. The percentage of anti-inflammatory T regulatory cells (Tregs) was lower in aorta from females (Figure 2E) and males (Figure 2F) on a HFD compared with a NFD.

**Figure 3:**
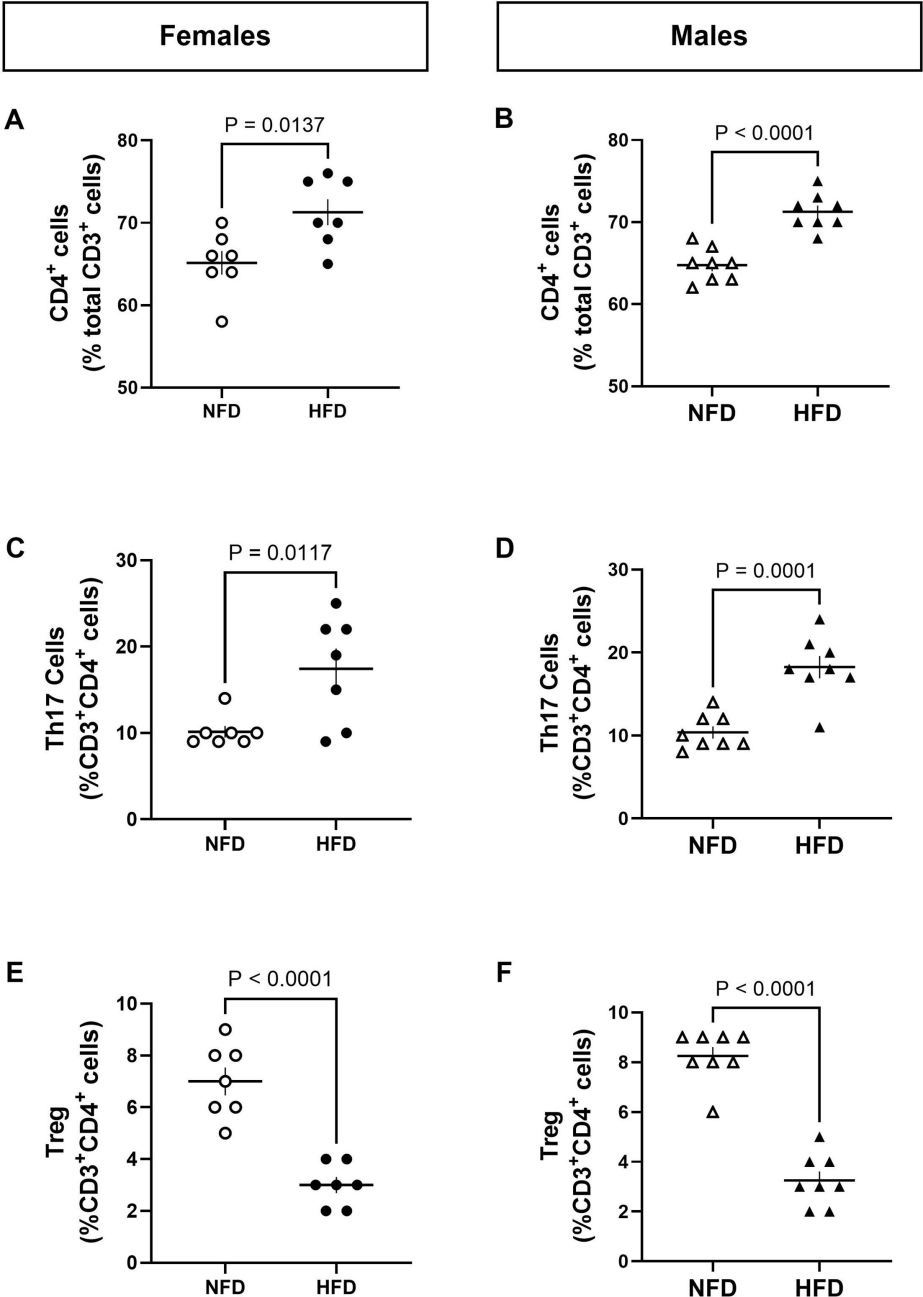
HFD promotes pro-inflammatory immune status in the kidney. Flow cytometry was used to measure the T cell profile in kidneys of female (left) and male (right) Dahl WT rats following ten weeks of normal-fat diet (NFD) or high-fat diet (HFD). The percentage of (**A-B**) CD4^+^, (**C-D**) Th17^+^ cells, and (**E-F**) Foxp3^+^ Tregs was measured. N values: **NFD-female** = 7, **NFD-male** = 8, **HFD-female** = 7, **HFD-male** = 8. Between-group differences were calculated via t-tests. Values are presented as mean ± SEM. HFD, high-fat diet; NFD, normal-fat diet; Treg, T regulatory cell.

T cells were also measured in the kidney. The percentage of renal CD3^+^ T cells was greater in males (**NFD-male**: 0.48 ± 0.04, **HFD-male**: 1.08 ± 0.13, *P*_t-test_ = 0.029) and females fed a HFD compared with a NFD (**NFD-female**: 0.43 ± 0.04, **HFD-female**: 0.86 ± 0.07, *P*_t-test_ = 0.0004). Similarly, the percentage of renal CD4^+^ T cells and Th17 cells was greater (Figure 3A–D), while the percentage of renal Tregs was lower (Figure 3E and F) following HFD treatment compared with rats consuming a NFD in both sexes.

### T cells directly contribute to HFD-induced hypertension in both female and male Dahl rats

**Figure 4:**
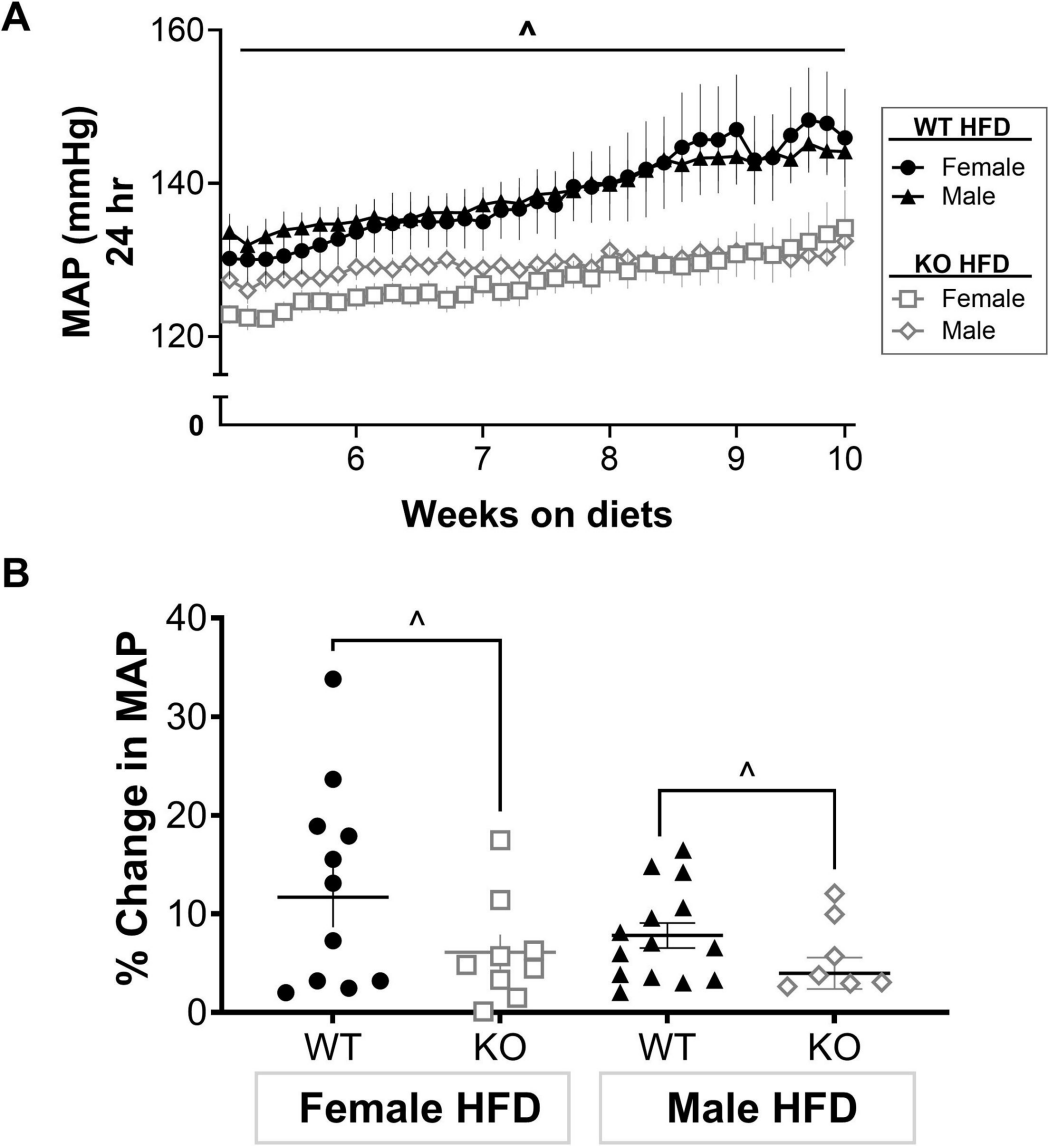
T cell KO blunted hypertension in both sexes. WT and T cell KO rats were placed on a HFD for ten weeks; telemeters were implanted four weeks into dietary treatment. (**A**) Mean arterial pressure (MAP) was measured in female and male rats. (**B**) The percentage change in MAP from weeks 5 to 10 of treatment was calculated. N values: **WT: HFD-female** = 12, **HFD-male** = 14; **T cell KO: HFD-female** = 10, **HFD-male** = 9. Between-group differences were calculated via two-way ANOVA. Data are presented as means ± SEM; ^ = main effect of genotype. HFD, high-fat diet; KO, knockout; MAP, mean arterial pressure; WT, wildtype.

To determine whether T cells mechanistically contribute to HFD-induced hypertension, female and male WT and CD247 KO Dahl rats were placed on a HFD for ten weeks, starting at five weeks of age. MAP was measured from five to ten weeks of HFD treatment via telemetry. While MAP increased over the dietary treatment period in both sexes, MAP was lower in KO vs. WT rats throughout the treatment (*P*_Genotype_ < 0.05, Figure 4A), directly demonstrating a role for T cells in HFD-induced hypertension. Since MAP was lower in KO rats and BP increased over time in all groups, the percentage change in MAP was calculated. WT rats on a HFD had greater increases in MAP vs. KO, regardless of sex (P_Interaction_ = 0.68, *P*_Sex_ = 0.17, and *P*_Genotype_ = 0.03, Figure 4B).

When comparing BP in WT females and males on a HFD, the increase in MAP was greater in females (mmHg: **HFD-female** = 11.69 ± 3.06; **HFD-male** = 7.80 ± 1.27). However, this did not reach statistical significance (*P*_t-test_ = 0.23). The increase in MAP was also greater in female KO vs. male KO on HFD (mmHg: **HFD-female** = 6.12 ± 1.78; **HFD-male** = 3.98 ± 1.59), but again this did not reach statistical significance (*P*_t-test_ = 0.38). MAP was higher in WT vs. KO rats on a NFD in both females and males (Supplementary Figure S2) with males tending to have higher MAP than females. These data suggest that T cells play a role in BP control under both NFD and HFD conditions.

### T cells do not contribute to HFD-induced weight gain in female or male Dahl rats

**Figure 5:**
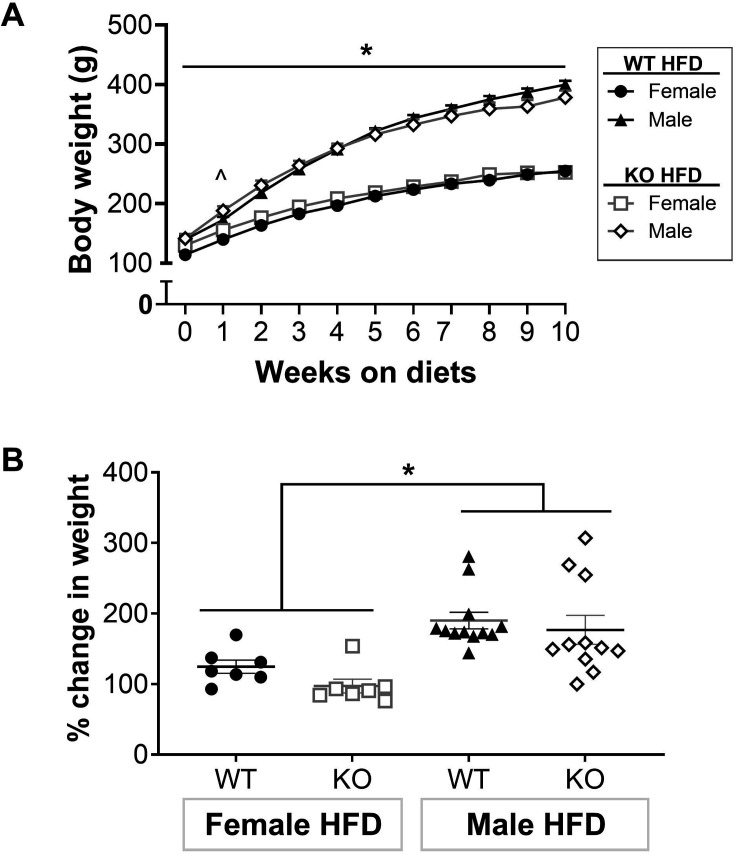
T cell KO did not change body weight compared with WT. (**A**) Rats were weighed weekly. (**B**) The percentage change in body weight was calculated. N values: **WT: HFD-female** = 7, **HFD-male** = 12; **T cell KO: HFD-female** = 7, **HFD-male** = 11. Between-group differences were calculated via two-way ANOVA. Values are presented as mean ± SEM. Symbols indicate *P*<0.05 for ^ = main effect of genotype, * = main effect of sex. HFD, high-fat diet; KO, knockout; WT, wildtype.

WT and CD247 KO rats were weighed weekly to determine if T cell KO affected HFD-induced weight gain. Body weight was greater in males vs. females regardless of genotype (*P*_Sex_<0.05, Figure 5A). While T cell KO rats of both sexes had blunted increases in body weight after one week of HFD (*P*_Genotype_ = 0.02), body weights were comparable at all other points. The percentage increase in body weight was also calculated. Males had greater increases in body weight vs. females regardless of genotype (*P*_Sex_<0.0001, Figure 5B). There was no difference in weight gain between WT and CD247 KO rats of either sex (*P*_Interaction_ = 0.65, *P*_Genotype_ = 0.21). Interestingly, WT rats on NFD were heavier than KO rats (*P*_Genotype_ < 0.05; Supplementary Figure S3), suggesting that KO rats gain more weight on a HFD vs. WT rats.

To confirm that T cell KO did not alter food intake, a subset of rats on HFD was placed in metabolic cages and fat consumption was calculated. Prior to the initiation of HFD, WT rats consumed more food (Supplementary Table S1) and kcal of fat (Supplementary Table S2) vs. KO rats regardless of sex. However, there were no differences in fat consumed between same sex in WT and KO rats during HFD treatment, suggesting that the difference in BP between WT and KO rats on HFD is not due to differences in fat consumption.

### T cell KO did not affect overall body fat, but attenuated increases in adipose tissue weights

**Figure 6:**
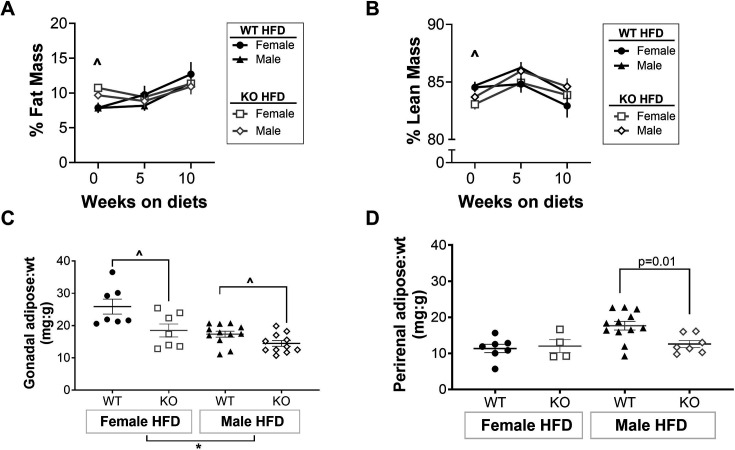
T cell KO attenuated the increases in adiposity. Nuclear magnetic resonance imaging (NMR) was used to measure body composition. (**A-B**) Following ten weeks of HFD, gonadal (**C**) and peri-renal (**D**) adipose tissues were isolated and weighed. N values: **WT: HFD-female** = 7, **HFD-male** = 12; **T cell KO: HFD-female** = 4–7, **HFD-male** = 7–11. Between-group differences were calculated via two-way ANOVA. Values are presented as mean ± SEM. Symbols indicate *P*<0.05 for ^ = main effect of genotype, * = main effect of sex. HFD, high-fat diet; KO, knockout; WT, wildtype.

Body composition was measured in WT and KO rats via NMR at baseline (0 weeks of diet) and following five and ten weeks of HFD. Prior to initiation of HFD, both female and male KO rats had greater body fat mass vs. WT (*P*_Interaction_ = 0.32, *P*_Sex_ = 0.30, and *P*_Genotype_< 0.0001, Figure 6A). After five weeks of HFD, there were no differences between WT and KO rats through the remainder of the study (*P*_Interaction_ > 0.05, *P*_Sex_> 0.05, and *P*_Genotype_ > 0.05). At baseline, both male and female WT rats had greater lean mass vs. KO rats (*P*_Interaction_ = 0.50, *P*_Sex_ = 0.30, and *P*_Genotype_ = 0.002, Figure 6B); however, there were no differences in lean mass between genotypes during HFD treatment (*P*_Interaction_ > 0.05, *P*_Sex_> 0.05, and *P*_Genotype_ > 0.05). These data suggest that CD247 KO did not alter HFD-induced changes in body mass composition in either sex.

Gonadal and perirenal adipose tissues were isolated following ten weeks on HFD. WT rats of both sexes had greater gonadal adipose tissue vs. KO rats (*P*_Interaction_ = 0.13, *P*_Genotype_ = 0.001, Figure 6C), and females of both genotypes had greater gonadal adipose tissue weight vs. males (*P*_Sex_ = 0.0001, Figure 6C). In contrast, males of both genotypes had greater perirenal adipose tissue weight vs. females (*P*_Sex_ = 0.02, *P*_Genotype_ = 0.13, Figure 6D). While T cell KO rats had lower gonadal adipose tissue weight vs. WT rats, perirenal adipose tissue weight was only lower in KO males (*P*_Interaction_ = 0.049, Figure 6D). These data suggest that while CD247 KO had an impact on individual adipose tissues on HFD, these changes were not sufficient to alter overall body mass composition in either sex. No genotype differences in fat mass, lean mass, or adipose tissue weights were observed in rats consuming a NFD (Supplementary Figure S4), suggesting that the lower adipose tissue weight in HFD groups is diet-specific.

### T cell KO did not affect vascular function or protein excretion in female Dahl rats on a HFD

**Figure 7:**
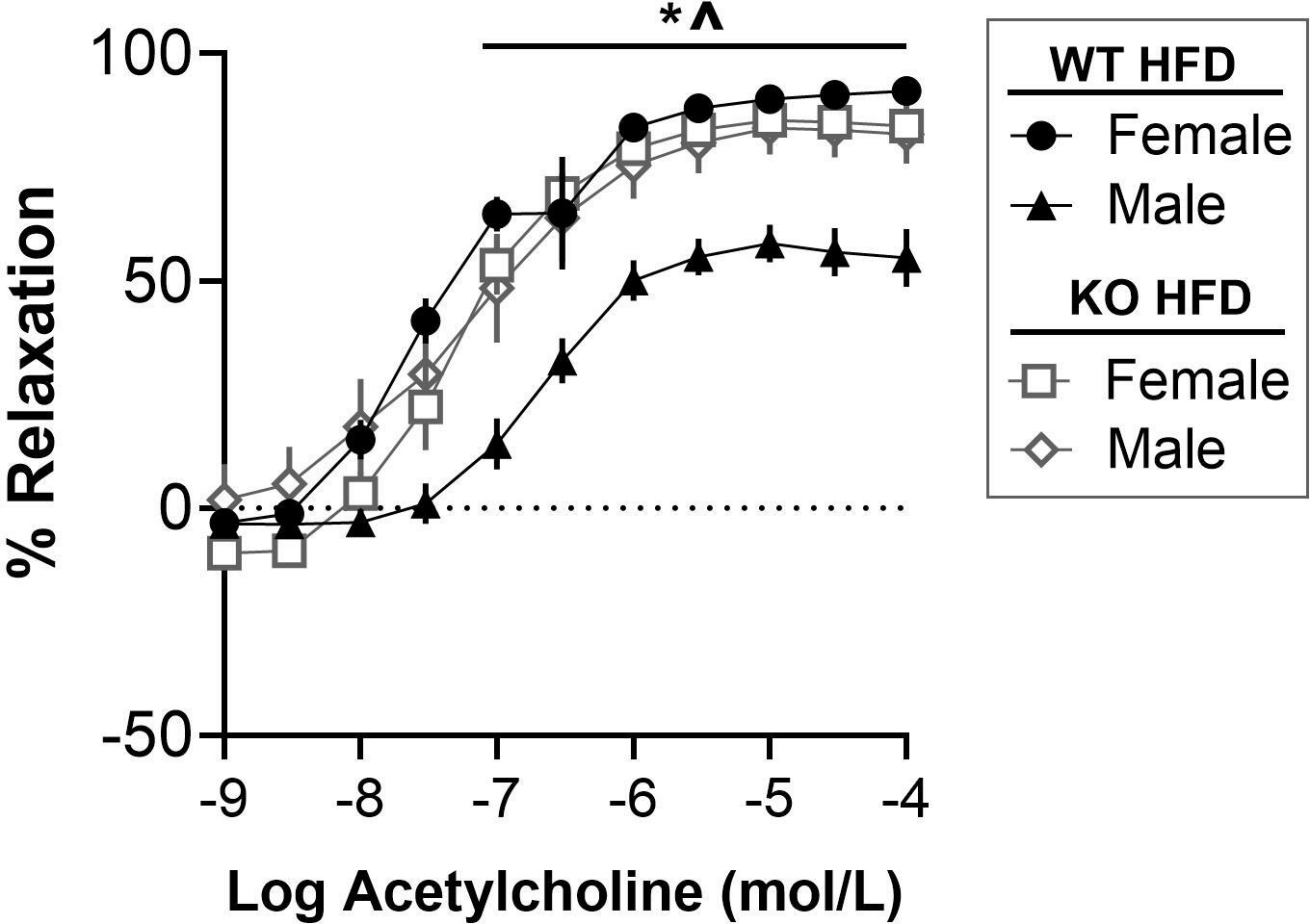
Male rats exhibit T cell-mediated increases in vascular dysfunction. Endothelial-dependent relaxation to acetylcholine was measured in aortic rings isolated from female and male WT and T cell KO rats on a HFD for ten weeks. N values: **WT: HFD-female** = 6, **HFD-male** = 5; **T cell** **KO:** **HFD-female** = 6, **HFD-male** = 6. Values are presented as mean ± SEM. Between-group differences were calculated via two-way ANOVA. Symbols indicate *P*<0.05 for ^ = main effect of genotype, * = main effect sex. HFD, high-fat diet; KO, knockout; WT, wildtype.

To begin exploring the potential mechanisms by which HFD and T cells regulate BP, vascular endothelial function in isolated aortic rings and urinary protein excretion were measured. WT HFD males had decreased Ach-induced endothelial-dependent relaxation compared with the other HFD groups (*P*<0.0001). Similarly, WT NFD males also exhibited less relaxation compared with all other NFD groups (*P*<0.0001; Figure 7 and Supplementary Figure S6). These data support a role for T cells to impair endothelial-dependent relaxation in males regardless of diet. The effect appears greater in the HFD-fed males vs. NFD-fed males, though this was not directly measured in the current study. However, relaxation was comparable in WT and KO females regardless of dietary treatment, indicating that T cells do not play a major role in maintaining endothelial-dependent relaxation in females like they do for males.

The 24-hour urinary protein excretion was measured in female and male WT and KO rats at the end of the ten-week HFD treatment. Male rats on a HFD had greater protein excretion vs. females, regardless of genotype (*P*_Interaction_ = 0.77, *P*_Sex_ = 0.0002, *P*_Genotype_ = 0.68). Protein excretion levels were (mg/day: **WT: HFD-female** = 59.3 ± 5.5, **HFD-male** = 134.8 ± 20.1; **T cell KO: HFD-female** = 47.3 ± 7.7, **HFD-male** = 132.8 ± 24.5). These data suggest that T cells did not contribute to sex difference in proteinuria during a HFD treatment.

## Discussion

The goal of the current study was to determine if T cells mediate HFD-induced increases in BP in male and female Dahl rats. While T cells have been implicated in the development of hypertension with a chronic HFD [16,24], no studies have directly tested the contribution of T cells to HFD-induced hypertension. The CD247 KO rat has been used to illustrate a direct role for T cells in salt-induced hypertension in male Dahl rats [13,15]. The main finding of the current study is that T cells significantly contribute to HFD-induced hypertension in female and male Dahl rats. Our data demonstrate that the attenuation of BP in CD247 KO rats on a HFD was unlikely due to differences in fat consumption, body weight, or overall changes in body fat content, supporting an inflammatory basis, rather than a metabolic one, for the development of HFD-induced hypertension.

Sex differences in BP in Dahl rats on NFD are largely abolished on a HFD, supporting a greater susceptibility of females vs. males to HFD-induced hypertension. While the clinical literature shows that women are more sensitive to salt- and fat-induced increases in BP vs. men [9,32–35], many animal models do not reflect this. There are numerous reports that female Dahl rats have an attenuated increase in BP to high salt vs. males [13,36] and many other studies showing that female rodents are protected from the negative cardiovascular complications of a HFD vs. males [37–39]. In contrast, consistent with previous studies, our findings show that female Dahl rats are not protected from HFD-induced increases in BP vs. males [24,29,30,35] and are in line with epidemiological evidence indicating enhanced susceptibility of women to the negative cardiovascular consequences of increases in adiposity [40,41]. Thus, HFD-fed female Dahl rats are an excellent model to mimic the human disease and understand contributing mechanisms.

Although many reports suggest that hypertensive males have a more pro-inflammatory T cell profile vs. females [13,25,26,31], HFD-induced hypertension appears to equally affect the inflammatory profile in the aorta and kidneys of female and male Dahl rats. Indeed, we speculate that the comparable increases in the pro-inflammatory status in female and male Dahl rats on a HFD underlie the lack of protection typically afforded females, as T cells have been implicated in hypertension [13,21–26]. We previously showed that greater Tregs in females drive the attenuated increase in BP with DOCA-salt vs. males [31] and Tregs protect young female mice from angiotensin II-induced increases in BP [42]. In addition, RAG1 KO mice, deficient in T and B cells, lack a sex difference in response to angiotensin II infusion [25,26], further supporting a key role for lymphocytes in sex difference in BP. Therefore, the finding that a chronic HFD results in comparable changes in T cells in both sexes may underlie the enhanced susceptibility of females to increases in BP.

CD247 KO rats had a lower BP than WT rats throughout the HFD treatment, although the difference in BP between the genotypes became greater with a longer duration of HFD. Commencing the HFD at five weeks of age precluded the measurement of BP for the full duration of dietary treatment since telemetry instrumentation of three-week-old rats is not feasible. Regardless, the lower BP in the KO rats suggests that T cells are important in the maintenance of BP. CD247 KO rats had a lower BP on NFD as well, although the magnitude of the difference was less than on HFD. Since CD247 KO rats exhibited an increase in BP on HFD, T cells are not the only factor mediating the increase in BP. Indeed, there were no significant differences in renal IL-1β and TNF-α in WT and KO rats. Spradley et al. demonstrated that treatment with mycophenolate mofetil (MMF) prevented HFD-induced hypertension [16], suggesting that T and B lymphocytes must be suppressed to observe a reversal of HFD-induced hypertension. Future studies will assess the contribution of additional immune cells to HFD-induced increases in BP.

Previous studies demonstrated that MMF decreased body weight in male Sprague Dawley rats [43], supporting a role for lymphocytes to regulate body weight. Consistent with this, male severe combined immunodeficiency mice, which lack B and T cells, have a lower body weight following a six-week HFD vs. C57BL/6 J mice [44,45]. CD247 KO did not change body weight or total fat mass in either sex on a HFD in the current study, although CD247 KO rats weighed less than WT rats on NFD. The genotype difference in weight was lost once placed on HFD, suggesting that CD247 KO rats gain more weight on HFD than WT rats and supporting the conclusion that the lower BP in CD247 KO rats cannot be attributed to lower body weight. Despite no differences in body weight or composition, there were regional differences in local fat depots with less white visceral adipose tissue in the CD247 KO, which has been linked to cardiovascular health [46,47]. It may be pertinent that KO males tended to have the least adipose tissue deposition and the smallest increases in BP with HFD. Future studies will include a more complete assessment of visceral and subcutaneous fat depots, adipokines, and potential sex differences in the metabolism of the HFD to further investigate a potential metabolic contribution to HFD-induced increases in BP. It is also important to note that despite having comparable HFD-induced increases in BP as males, females gained a lower amount of weight compared with males, supporting the epidemiological studies showing that cardiovascular health in women is more susceptible to changes in weight. In contrast with studies indicating that male mice display greater caloric intake and body weight gain than females when offered HFD [48], there were no differences in fat consumption between female and male Dahl rats in the current study.

To gain insight into the mechanisms driving HFD-induced increases in BP and the enhanced susceptibility of females, we examined vascular and renal dysfunction, both of which contribute to the development and maintenance of hypertension. Interestingly, we found that T cells contribute to endothelial dysfunction in males, but not females. Although BP increases to a similar degree in females and males during HFD treatment, females maintained endothelial function and had less proteinuria than males, suggesting that the enhanced susceptibility of females to HFD-induced hypertension is not mediated by greater vascular or renal injury. Future studies will measure vascular reactivity in other arteries and conduct a more in-depth assessment of kidney health in WT and CD247 KO rats fed a HFD and NFD.

There are limitations in the current study. BP was not measured throughout the whole dietary treatment. While future studies will delay the onset of HFD to a later age to allow collection of BP data for the full ten weeks, age is an important factor in diet-induced weight gain [28,49,50]. The diet was started soon after weaning to reflect early life consumption of a HFD. In the current study, only T cell percentages were measured, not T cell activation. However, since CD247 KO attenuated the development of hypertension, this concern is somewhat mitigated. In addition to the established sex differences in the distribution of adipose tissue, there are also other metabolic-related sex differences that affect adipose tissue such as lipoprotein lipase activity involved in triglyceride uptake and insulin sensitivity [51]. Future studies will investigate if HFD or CD247 KO changes additional metabolic parameters.

**Clinical perspectives**

- **Background as to why the study was undertaken:** There is increasing epidemiological evidence that women are more susceptible than men to increases in cardiovascular disorders, including hypertension, with increases in adiposity.
- **Brief summary of the results:** This study provides the first direct evidence that T cells contribute to high-fat diet (HFD)-induced increases in blood pressure (BP) in both sexes.
- **Potential significance of the results to human health and disease:** Since chronic consumption of HFD contributes to hypertension, and measures to limit the intake of foods high in fat are difficult, it is imperative to understand how HFD affects BP in both sexes.

## Supplementary Material

Online supplementary material 1

## Data Availability

We agree to make any materials, data, code, and associated protocols available. All data associated with this study are included in the manuscript and associated Supplement.

## Open Access

This article has been published open access under our Subscribe to Open programme, made possible through the support of our subscribing institutions, learn more here: https://portlandpress.com/pages/open_access_options_and_prices#conditional

## Abbreviations

Ach: acetylcholine
BMI: body mass index
BP: blood pressure
CVD: cardiovascular disease
FOXP3: forkhead box P3
HFD: high-fat diet
KO: knockout
NFD: normal-fat diet
NMR: nuclear magnetic resonance
PE: phenylephrine
Tregs: T regulatory cells
WT: wildtype
