# Supplementary material for: T cell knockout attenuates HFD-induced increases in blood pressure in female and male Dahl rats
Source: Clin Sci (Lond). 2025 Sep 9;139(17):941–54. doi: 10.1042/CS20257273 (PMC12599257; doi:10.1042/CS20257273)
Supplement: Online supplementary material 1 [file cs-139-17-CS20257273-s001.docx]

**SUPPLEMENT TO:**

**T cell Knockout Attenuates HFD-Induced Increases in Blood Pressure in Female and Male Dahl Rats**

Lindsey A. Ramirez, PhD^1^; Elizabeth Snyder, MD ^1^; Riyaz Mohamed, PhD^1^; Justine M. Abais-Battad, PhD^1^; Hannah R. Goldey-Boswell^1^, John Henry Dasinger, PhD^1^; David L. Mattson, PhD^1^; Mike W. Brands, PhD^1,^ Babak Baban, PhD^2;^ Ahmed Elmarakby, PhD^2^; Michael J. Ryan, PhD^3,4^ and Jennifer C. Sullivan, PhD^1^

Medical College of Georgia at Augusta University, Augusta, GA: ^1^Department of Physiology, ^2^Department of Oral Biology, ^3^ Columbia VA Health Care System, Columbia, SC and ^4^University of South Carolina School of Medicine, Columbia, SC.

**Short Title: T cell KO attenuates hypertension with a high fat diet**

**Corresponding Author:**

Jennifer C. Sullivan, PhD

[jensullivan@augusta.edu](mailto:jensullivan@augusta.edu)

1459 Laney Walker Blvd

Augusta University

Augusta, GA 30912

Phone: 706-721-9796

Fax: 706-721-7661

**METHODS**

**Animals**


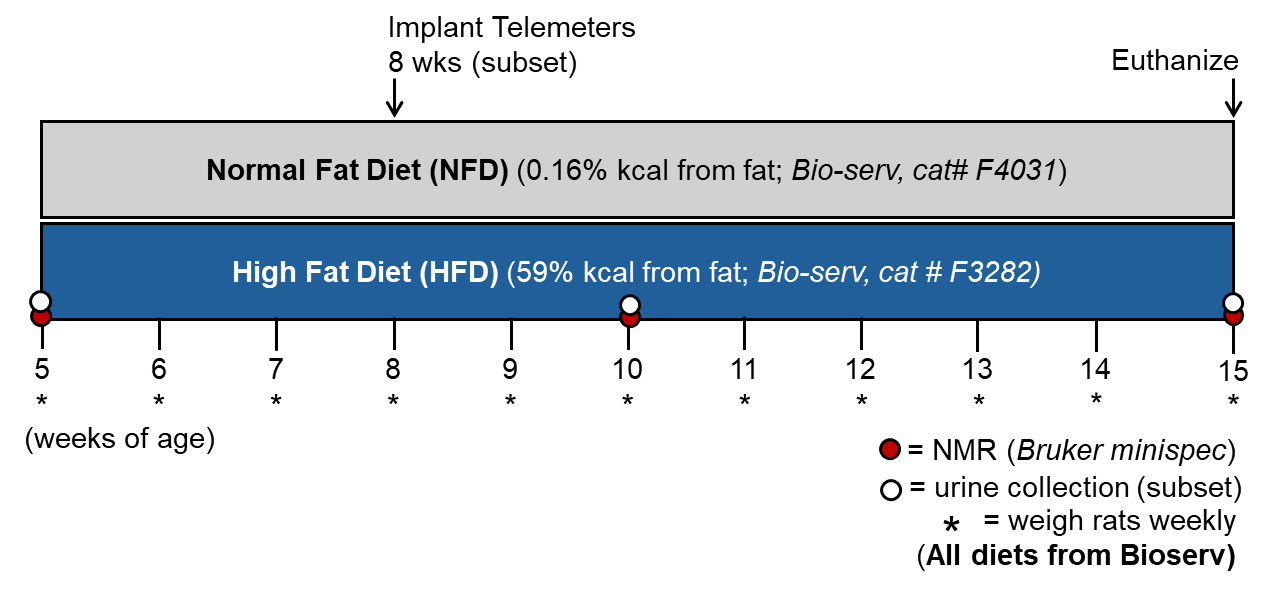
All animal experiments were approved by the Augusta University Institutional Animal Care and Use Committee and conducted in accordance with the National Institutes of Health Guide for the Care and Use of Laboratory Animals. Initial studies included male and female wildtype (WT) Dahl rats from a colony maintained at Augusta University originally obtained from the Medical College of Wisconsin (SS/JrHsdMcwi). Rats were maintained in temperature-and humidity-controlled rooms on a 12-hour light: dark cycle and maintained on an AIN purified diet containing 0.4% NaCl (Dyets, cat# 113755GI). Rats were weaned at ~21 days of age and maintained on the AIN purified diet. At 5 weeks of age, rats were randomized to receive either a normal fat diet (NFD) with 0.16% kcal from fat (*Bio-serv, cat# F4031, https://www.bio-serv.com/pdf/F4031.pdf*) or a HFD with 59% kcal from fat (major fat component is lard, *Bio-serv, cat # F3282, https://www.bio-serv.com/pdf/F3282_S3282.pdf*) for 10 weeks. Note, both NFD and HFD contain equal amounts of NaCl, 0.4%. Food and water were available ad libitum.

To directly assess the contribution of T cells to HFD-induced increases in BP and adiposity, additional studies included WT female and male Dahl rats and CD247 knockout Dahl rats (T cell KO; **Supplementary Figure 1**). The CD247 KO was originally obtained from the Medical College of Wisconsin. Rats were bred in the barrier facility using homozygous breeding pairs. Male and female wildtype (WT) and CD247 KO Dahl rats were weaned at ~21 days of age and maintained on the AIN purified diet. At 5 weeks of age, rats were placed on a normal fat diet (NFD) with 0.16% kcal from fat (*Bio-serv, cat# F4031, https://www.bio-serv.com/pdf/F4031.pdf*) for 10 weeks. Since the primary question of the current study was the impact of T cells on HFD-induced increases in BP and adiposity in females and males, rats on NFD and HFD were not directly compared.

**Renal Cytokine Measurements**

Kidneys were weighed and homogenized based on 1 to 10 ratio in 1X RIPA lysis buffer (Cat # 89901, Thermo Scientific, Rockford, IL) in the presence of a protease (Halt protease inhibitor cocktail, Cat # 87785; Thermo Scientific, Rockford, IL) and phosphatase inhibitor cocktails (Cat #  P5726; Sigma Aldrich). The homogenates were centrifuged at 3,000 *g* for 10 minutes, and the supernatant was collected. Protein concentrations were determined by Pierce BCA assay kit (Thermo Scientific, 1 to 100 dilution) using BSA as standard. Renal homogenates were diluted 1:2 in sample buffer and then used to determine renal TNF-α and Il-1β using commercial ELISA kits from (R&D system for TNF-α Cat #RTA-001, and ThermoFisher for IL-1β Cat # BMS630). Values were measured as pg/ml and reported as pg/mg protein.

**RESULTS**

**T cell KO lowers BP but does not alter BP responses to NFD**

At multiple times throughout the NFD treatment, MAP was greater in males compared to females regardless of genotype (P_Sex_<0.05, **Supplementary Figure 2A**). Similar to rats on a HFD, MAP was greater in WT rats on a NFD vs. KO rats (P_Genotype_<0.05). The percentage change in MAP was calculated. While MAP increased over time with age, the percent increase was comparable among the different groups (**wt:** **NFD Female**= 4.47 ± 1.7, **NFD Male**= 4.70 ± 1.4; **t cell ko:**  **NFD Female**= 3.78 ± 1.4, **NFD Male**= 2.71 ± 1.30). Thus, there were no statistical differences between any of the groups (P_Interaction_=0.66, P_Sex_=0.78, P_Genotype_=0.37, **Supplementary Figure 2B**). These data suggest that T cells play a role in maintaining BP regardless of dietary treatment.

### WT rats gain more body weight over time on NFD than T cell KO rats

Throughout the dietary treatment, males were heavier than females on NFD regardless of genotype (P_Sex_<0.05, **Supplementary** **Figure 3A**). WT rats weighed more than KO rats during the 4^th^, 6^th^, and 8-10^th^ weeks of treatment (P_Genotype_<0.05). We further calculated the percent increase in body weight during the 10-week NFD treatment. The increase in body weight was greater in males compared to females regardless of genotype (P_Interaction_>0.05, P_Sex_<0.05, **Supplementary Figure 3B**). These data suggest that T cells contribute to the maintenance of body weight in both sexes.

Female and male WT and KO Dahl rats were placed in metabolic cages for 24 hours at 0, 5, and 10 weeks of treatment with dietary treatment to assess amount of food ingested, water consumed, and urine excreted. In the NFD groups, KO rats consumed less food and fewer kcal of fat at baseline and following 5 and 10 weeks of NFD treatment vs. WT rats (**Supplementary Tables 3 and 4**). Water intake was comparable among all rats at most time points, although females drank less water at baseline than males and as a result urine excretion was less.

**T cell KO did not affect overall body fat distribution on a NFD**

Female rats had greater fat mass and lower lean mass compared to males following 5 weeks of NFD treatment regardless of genotype (P_sex_<0.06, **Supplementary Figure 4A & 4B**). Similar to rats fed a HFD, females had greater gonadal adipose tissue weight compared to males on NFD (P_Interaction_=0.55, P_Sex_=0.003, P_Genotype_=0.21, **Supplementary Figure 4C**) and males had greater perirenal adipose tissue weight compared to females on NFD (P_Interaction_=0.42, P_Sex_=0.045, P_Genotype_=0.07, **Supplementary Figure 4D**). However, in contrast to the effect seen with HFD, T cell KO did not alter adipose tissue weights in rats fed a NFD.

**T cell KO does not significantly alter renal IL1-β or TNF-α**

Renal interleukin (IL)-1β and tumor necrosis factor (TNF)-α were measured in renal homogenates from male and female WT and CD247KO rats following a 10-week NFD or HFD. Renal IL-1β was greater in males than females on both a NFD (P_Sex_=0.0001) and HFD (P_Sex_=0.0002; **Supplementary Figure 5A and 5B**). There were no differences in renal IL-1β in WT and CD247 KO rats (NFD: P_Interaction_=0.18, P_Genotype_=0.84; HFD: P_Interaction_=0.77, P_Genotype_=0.68). There were no significant differences in renal TNF-α in rats maintained on a NFD (P_Interaction_=0.59, P_Sex_=0.40, P_Genotype_=0.21; **Supplementary Figure 5C**) or a HFD (P_Interaction_=0.087, P_Sex_=0.50, P_Genotype_=0.068; **Supplementary Figure 5D**), although there was a trend for TNF-α to be lowed in female KO on HFD vs. WT on HFD.

**Male rats exhibit T cell-mediated increases in vascular dysfunction and T cells contribute to proteinuria regardless of diet**

Vascular endothelial function in isolated aortic rings and urinary protein excretion were measured. Interestingly, endothelial dependent relaxation to acetylcholine was less in WT male on NFD compared to T cell KO males and females of both genotypes (P<0.0001) (**Supplementary Figure 6**). These data support a role for T cells in decreasing endothelial dependent relaxation in males vs. females regardless of the diet which may contribute to the increase is BP on a HFD in males. Ach relaxation was comparable in WT and T cell KO females regardless of dietary treatment (**Supplementary Figure 6)**, indicating that changes in vascular function cannot explain the enhanced BP sensitivity to a HFD in females.

24 hour urinary protein excretion was measured in female and male WT and CD247 KO rats at the end of the 10-week NFD treatment. Male rats on a NFD had greater protein excretion vs. females, regardless of genotype (P_Interaction_=0.18, P_Sex_=0.0001, P_Genotype_=0.84). Protein excretion levels were (mg/day: **wt: NFD Female**= 58.7 ± 7.7, **NFD Male**= 117.3 ± 15.5.1; **t cell ko: NFD Female**= 42.6 ± 9.1, **NFD Male**= 142.5 ± 15.1). These data suggest greater BP in NFD males vs. females coincided with greater proteinuria regardless of genotype.


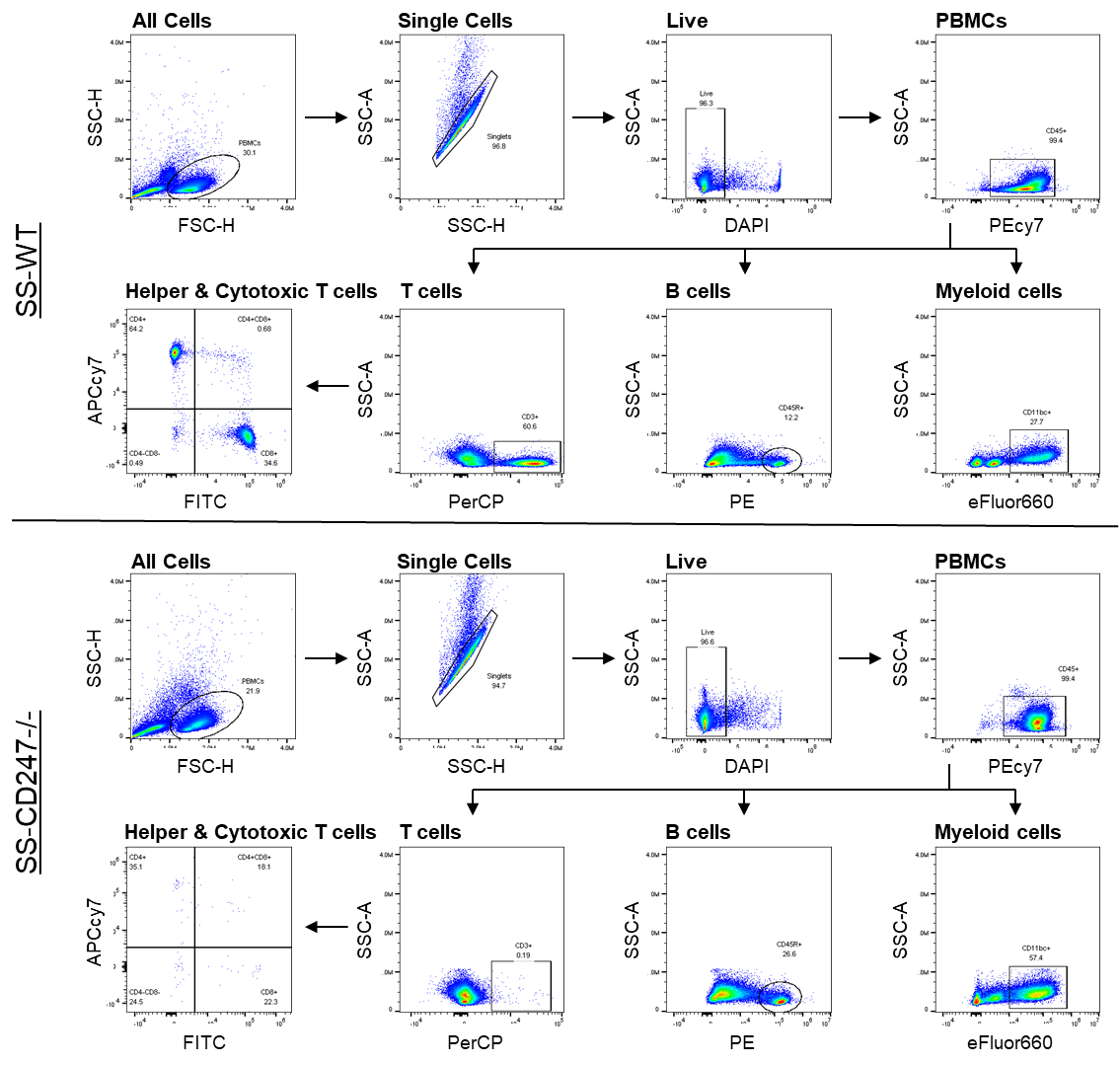


**Supplemental Figure 1**. Flow cytometric validation of T cell knockout in the circulation of SS-CD247-/- rats. Representative flow cytometry gating strategy used to identify CD45+ leukocytes, CD3+ T cells, CD4+ T helper cells, CD8+ cytotoxic T cells, CD45R+ B cells, and CD11b/c+ myeloid cells in PBMCs isolated from the blood.

**Supplementary Figure 2**. **T cell KO lowers BP but does not alter BP responses to NFD.** WT and T cell KO rats were placed on a NFD for 10 weeks; telemeters were implanted 4 weeks into dietary treatment. **(A)** Mean arterial pressure (MAP) was measured in female and male rats. **(B)** The percentage change in MAP from weeks 6 to 10 of treatment was calculated. N values: **wt: NFD-Female**=9, **NFD-Male**=9; **t cell ko:** **NFD-Female**=8, **NFD-Male**=7. Between group differences were calculated via 2-way ANOVA. Data are presented as means ± SEM; ^=main effect of genotype, *=main effect of sex.

**Supplementary Figure 3**. **T cell KO did not affect overall body fat distribution on a NFD. (A)** Rats were weighed weekly. **(B)** The percentage change in body weight was calculated. N values: **wt: NFD Female**=9, **NFD-Male**=9, **T cell ko:** **NFD-Female**=7, **NFD-Male**=10. Between group differences were calculated via 2-way ANOVA. Values are presented as mean ±SEM. Symbols indicate P<0.05 for ^ =main effect of genotype, *=main effect of sex.

**Supplementary Figure 4. T cell KO did not alter adiposity on a NFD.** Nuclear magnetic resonance imaging (NMR) was used to measure body composition **(A-B)** Following 10 weeks of HFD, gonadal **(C)** and peri-renal **(D)** adipose tissue were isolated and weighed. N values for body composition and gonadal adipose tissue: **wt**: **NFD-Female**=9, **NFD-Male**=8-9; **t cell ko**: **NFD-Female**=7, **NFD-Male**=10. N values for peri-renal adipose tissue: **wt**: **NFD-Female**=9, **NFD-Male**=9; **t cell ko**: **NFD-Female**=4, **NFD-Male**=6**.** Between group differences were calculated via 2-way ANOVA. Values are presented as mean ±SEM. Symbols indicate P<0.05 for *=main effect of sex.


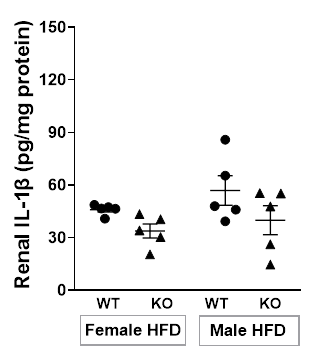

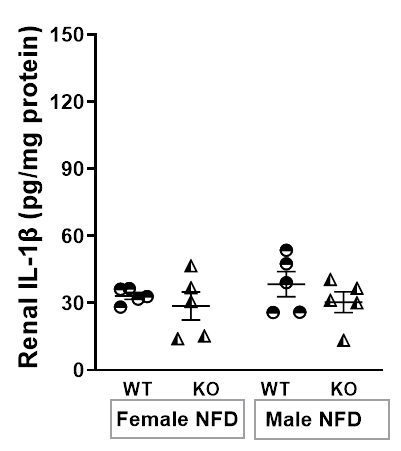


**B.**

**A.**


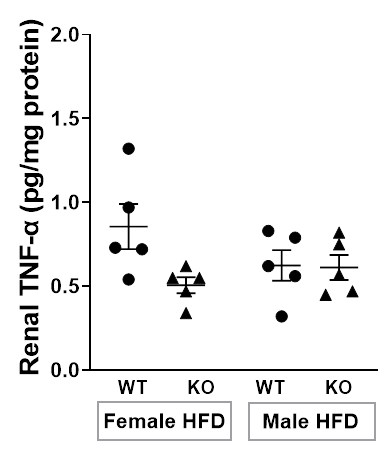

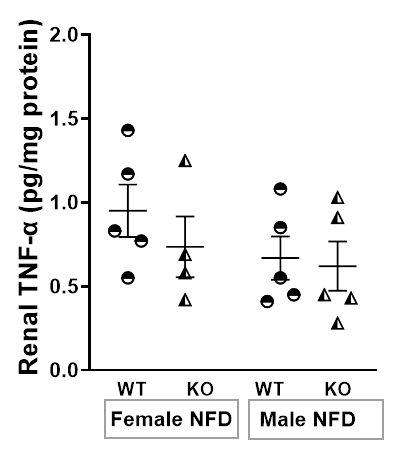


**D.**

**C.**

**Supplementary Figure 5. T cell KO does not significantly alter renal IL1-β or TNF-α.** IL-1β (A-B) and TNF-α (C-D) were measured by ELISA in renal homogenates from female and male WT and CD247 KO rats following 10 weeks of NFD or HFD. N values for IL-1β measurements: **wt**: **NFD-Female**=5, **NFD-Male**=5; **t cell ko**: **NFD-Female**=5, **NFD-Male**=5. N values for TNF-α measurements: **wt**: **NFD-Female**=5, **NFD-Male**=5; **t cell ko**: **NFD-Female**=4, **NFD-Male**=5**.** Between group differences were calculated via 2-way ANOVA. Values are presented as mean ±SEM.

**Supplementary Figure 6.** **Male rats exhibit T cell-mediated increases in impaired vascular function vs. females**. Endothelial-dependent relaxation to acetylcholine was measured in aortic rings isolated from female and male WT and T cell KO rats on a NFD for 10 weeks. N values: **wt**: **NFD-Female**=5, **NFD-Male**=5; **t cell ko**: **NFD-Female**=6, **NFD-Male**=4. Values are presented as mean ±SEM. Between group differences were calculated via 2-way ANOVA. Symbols indicate P<0.05 for ^ =main effect of genotype, *=main effect sex.

| **Urine excretion (mL/day)** | | | | | | | | | | | | |
| --- | --- | --- | --- | --- | --- | --- | --- | --- | --- | --- | --- | --- |
|  | | **Female** | | | | | **Male** | | | | |  |
|  | | **WT-HFD** | | | **KO-HFD** | | **WT-HFD** | | | **KO-HFD** | | p value |
| **Baseline**  **(Week 0)** | | 7.05 ± 0.9 | | 5.87 ± 1.4 | | | 7.92 ± 1.0 | 9.05 ± 1.3 | | | | P_Interaction_=0.37  P_Sex_=0.12  P_Genotype_=0.98 |
| **Week 5** | | 7.76 ± 0.9 | | 6.26 ± 2.1 | | | 5.65 ± 1.2 | 8.64 ± 1.0 | | | | P_Interaction_=0.10  P_Sex_=0.91  P_Genotype_=0.57 |
| **Week 10** | | 14.41 ± 5.4 | | 6.76 ± 2.1 | | | 9.75 ± 3.6 | 7.97 ± 0.7 | | | | P_Interaction_=0.98  P_Sex_=0.70  **P_Genotype_=0.02** |
| **Food consumption (g/day)** | | | | | | | | | | | | |
|  | **Female** | | | | | **Male** | | | | |  | |
|  | **WT-HFD** | | **KO-HFD** | | | **WT-HFD** | | | **KO-HFD** | | p value | |
|  |  | |  | | |  | | |  | |  | |
| **Baseline**  **(Week 0)** | 17.87 ± 2.1 | | 13.75 ± 0.9 | | | 20.19 ± 1.7 | | | 15.49 ± 1.0 | | P_Interaction_=0.85  P_Sex_=0.20  **P_Genotype_=0.009** | |
| **Week 5** | 17.36 ± 2.9 | | 13.62 ± 1.6 | | | 14.72 ± 1.0 | | | 14.75 ± 1.0 | | P_Interaction_=0.26  P_Sex_=0.64  P_Genotype_=0.26 | |
| **Week 10** | 11.98 ± 0.9 | | 15.10 ± 2.2 | | | 12.96 ± 1.3 | | | 12.22 ± 0.9 | | P_Interaction_=0.18  P_Sex_=0.50  P_Genotype_=0.40 | |
| **Water intake (mL/day)** | | | | | | | | | | | | |
|  | **Female** | | | | | **Male** | | | | |  | |
|  | **WT-HFD** | | **KO-HFD** | | | **WT-HFD** | | | **KO-HFD** | | p value | |
| **Baseline**  **(Week 0)** | 18.60 ± 2.3 | | 16.18 ± 1.7 | | | 17.79 ± 1.4 | | | 21.70 ± 2.9 | | P_Interaction_=0.21  P_Sex_=0.35  P_Genotype_=0.76 | |
| **Week 5** | 20.30 ± 2.0 | | 15.22 ± 2.8 | | | 25.39 ± 1.9 | | | 19.59 ± 2.2 | | P_Interaction_=0.88  P_Sex_=0.05  **P_Genotype_=0.03** | |
| **Week 10** | 26.05 ± 6.5 | | 18.00 ± 1.8 | | | 29.40 ± 3.1 | | | 20.40 ± 1.0 | | P_Interaction_=0.88  P_Sex_=0.39  **P_Genotype_=0.02** | |
|  |  | |  | | |  | | |  | |  | |
|  |  | |  | | |  | | |  | |  | |

**Supplementary Table 1**. **Metabolic cage data for HFD rats**. Female and male WT and T cell KO Dahl rats were placed in metabolic cages for 24 hours at 0, 5, and 10 weeks of treatment with high fat diet (HFD). The amount of food ingested in g/day, water intake in mL/day, and urine excreted in mL/day were measured and reported. Between group differences were calculated via 2-way ANOVA. Values are presented as mean ±SEM. N values: **wt**: **HFD Female**=4-5, **HFD Male**=5-8; **t cell ko: HFD Female**= 5, **HFD Male**= 7-9.

|  | | **WT-HFD** | **KO-HFD** | | **WT-HFD** | **KO-HFD** | | **p** |
| --- | --- | --- | --- | --- | --- | --- | --- | --- |
| **Caloric consumption (Kcal/day)** | **Female** | | | **Males** | | |  | |
| **Baseline**  **(Week 0)** | | n= 5 | n= 4-5 | | n= 8 | n= 7-9 | |  |
|  |  | 57.89 ± 6.7 | 44.56 ± 2.9 | | 65.41 ± 5.5 | 50.19 ± 3.3 | | P_Interaction_=0.85  P_Sex_=0.19  **P_Genotype_=0.01** |
| **Week 5** | | 56.25 ± 9.3 | 44.13 ± 5.1 | | 47.69 ± 3.2 | 47.79 ± 3.1 | | P_Interaction_=0.26  P_Sex_=0.64  P_Genotype_=0.26 |
| **Week 10** | | 38.82 ± 2.99 | 48.92 ± 7.2 | | 41.98 ± 4.3 | 39.60 ± 3.1 | | P_Interaction_=0.18  P_Sex_=0.50  P_Genotype_=0.40 |

**Supplementary Table 2. T** **cell KO did not affect the kcal fat consumed.** Female and male WT and T cell KO Dahl rats were placed in metabolic cages for 24 hours at 0, 5, and 10 weeks of treatment with high fat diet (HFD). The amount of food ingested was measured in g/day and kcals/day were calculated. Between group differences were calculated via 2-way ANOVA. Values are presented as mean ±SEM. **wt**: **HFD Female**=5, **HFD Male**=8; **t cell ko: HFD Female**= 4-5, **HFD Male**= 7-9.

| **Urine excretion (mL/day)** | | | | | | | | | | | | |
| --- | --- | --- | --- | --- | --- | --- | --- | --- | --- | --- | --- | --- |
|  | | **Female** | | | | | **Male** | | | | |  |
|  | | **WT-NFD** | | | **KO-NFD** | | **WT-NFD** | | | **KO-NFD** | | p value |
| **Baseline**  **(Week 0)** | | 9.73 ± 1.3 | | 7.5 ± 2.1 | | | 12.53 ± 2.2 | 14.27 ± 2.0 | | | | P_Interaction_=0.34  **P_Sex_=0.03**  P_Genotype_=0.90 |
| **Week 5** | | 13.35 ± 2.3 | | 9.3 ± 2.3 | | | 14.06 ± 3.3 | 13.10 ± 2.2 | | | | P_Interaction_=0.57  P_Sex_=0.41  P_Genotype_=0.37 |
| **Week 10** | | 16.31 ± 3.5 | | 11.38 ± 2.4 | | | 15.48 ± 3.5 | 12.20 ± 1.7 | | | | P_Interaction_=0.76  P_Sex_=0.99  P_Genotype_=0.15 |
| **Food consumption (g/day)** | | | | | | | | | | | | |
|  | **Female** | | | | | **Male** | | | | |  | |
|  | **WT-NFD** | | **KO-NFD** | | | **WT-NFD** | | | **KO-NFD** | | p value | |
|  |  | |  | | |  | | |  | |  | |
| **Baseline**  **(Week 0)** | 16.00 ± 2.6 | | 12.73 ± 0.7 | | | 18.85 ± 1.0 | | | 13.56 ± 0.7 | | P_Interaction_=0.46  P_Sex_=0.18  **P_Genotype_=0.004** | |
| **Week 5** | 16.19 ± 1.2 | | 13.66 ± 0.9 | | | 17.11 ± 0.6 | | | 14.90 ± 0.7 | | P_Interaction_=0.85  P_Sex_=0.22  **P_Genotype_=0.01** | |
| **Week 10** | 15.90 ± 2.0 | | 10.41 ± 1.2 | | | 17.98 ± 1.2 | | | 16.34 ± 0.4 | | P_Interaction_=0.13  **P_Sex_=0.004**  **P_Genotype_=0.008** | |
| **Water intake (mL/day)** | | | | | | | | | | | | |
|  | **Female** | | | | | **Male** | | | | |  | |
|  | **WT-NFD** | | **KO-NFD** | | | **WT-NFD** | | | **KO-NFD** | | p value | |
| **Baseline**  **(Week 0)** | 19.51 ± 1.7 | | 18.28 ± 1.5 | | | 24.46 ± 2.6 | | | 24.71 ± 2.0 | | P_Interaction_=0.74  **P_Sex_=0.02**  P_Genotype_=0.82 | |
| **Week 5** | 24.43 ± 3.1 | | 19.08 ± 2.8 | | | 26.81 ± 2.8 | | | 22.62 ± 2.0 | | P_Interaction_=0.83  P_Sex_=0.29  P_Genotype_=0.10 | |
| **Week 10** | 27.12 ± 4.2 | | 21.72 ± 3.6 | | | 33.85 ± 6.6 | | | 25.06 ± 2.4 | | P_Interaction_=0.71  P_Sex_=0.28  P_Genotype_=0.13 | |
|  |  | |  | | |  | | |  | |  | |
|  | | | | | | | | | | | | |

**Supplementary Table 3**. **Metabolic cage data for NFD rats**. Female and male WT and T cell KO Dahl rats were placed in metabolic cages for 24 hours at 0, 5, and 10 weeks of treatment with normal fat diet (NFD). The amount of food ingested in g/day, water intake in mL/day, and urine excreted in mL/day were measured and reported. Between group differences were calculated via 2-way ANOVA. Values are presented as mean ±SEM N values: **wt**: **NFD Female**=5, **NFD Male**=5-7; **t cell ko**: **NFD Female**= 4-5, **NFD Male**= 6-7.

|  | **WT-NFD** | | **KO-NFD** | | **WT-NFD** | **KO-NFD** | | **p** |
| --- | --- | --- | --- | --- | --- | --- | --- | --- |
| **Caloric consumption (Kcal/day)** | | **Female** | | **Males** | | |  | |
| **Baseline**  **(Week 0)** | n= 5 | | n= 4-5 | | n= 6-7 | n= 6-7 | |  |
|  | 13.74 ± 1.4 | | 8.15 ± 0.5 | | 12.06 ± 0.6 | 8. 7 ± 0.4 | | P_Interaction_=0.16  P_Sex_=0.46  **P_Genotype_<0.0001** |
| **Week 5** | 10.36 ± 0.8 | | 8.74 ± 0.6 | | 10.95 ± 0.4 | 9.54 ± 0.4 | | P_Interaction_=0.85  P_Sex_=0.22  **P_Genotype_=0.01** |
| **Week 10** | 10.18 ± 1.3 | | 6.66 ± 0.8 | | 11.50 ± 0.8 | 10. 46 ± 0.3 | | P_Interaction_=0.13  **P_Sex_=0.004**  **P_Genotype_=0.008** |

**Supplementary Table 4**. **Kcal of fat consumed in NFD rats.** Female and male WT and T cell KO Dahl rats were placed in metabolic cages for 24 hours at 0, 5, and 10 weeks of treatment with normal fat diet (NFD). The amount of food ingested in g/day was measured and kcal/day of fat ingested was calculated. Between group differences were calculated via 2-way ANOVA. Values are presented as mean ±SEM. **wt**: **NFD Female**=5, **NFD Male**=6-7; **t cell ko**: **NFD Female**= 4-5, **NFD Male**= 6-7.

**Supplementary Table 5**. Major Resources Table for flow cytometry antibodies.

| **Antibody Name** | **Company** | **Catalog #** | **RRID** |
| --- | --- | --- | --- |
| CD3- PerCP | Invitrogen | 46-0030-82 | AB_2573666 |
| CD4-FITC | BD Pharmingen | 554837 | AB_395547 |
| FOX P3-APC | Invitrogen | 17-5773-82 | AB_469457 |
| CD3 PE | Invitrogen | 12-0030-82 | AB_465493 |
| CD3 FITC | Invitrogen, eBioscience | 11-0030-82 | AB_464878 |
| ROR gamma PE | R&D systems | IC 6006P | [AB_2044720](http://antibodyregistry.org/AB_2044720) |
| CD3 APC | BD Pharmingen | 557030 | AB_398622 |
